# Supplementary material for: High-throughput small molecule screen identifies inhibitors of microsporidia invasion and proliferation in C. elegans
Source: Nat Commun. 2022 Sep 26;13:5653. doi: 10.1038/s41467-022-33400-y (PMC9513054; doi:10.1038/s41467-022-33400-y)
Supplement: Supplementary file 1 — Supplementary Information [file 41467_2022_33400_MOESM1_ESM.pdf]

Supplementary Information

High-throughput small molecule screen identifies inhibitors of microsporidia invasion and proliferation in *C. elegans*.

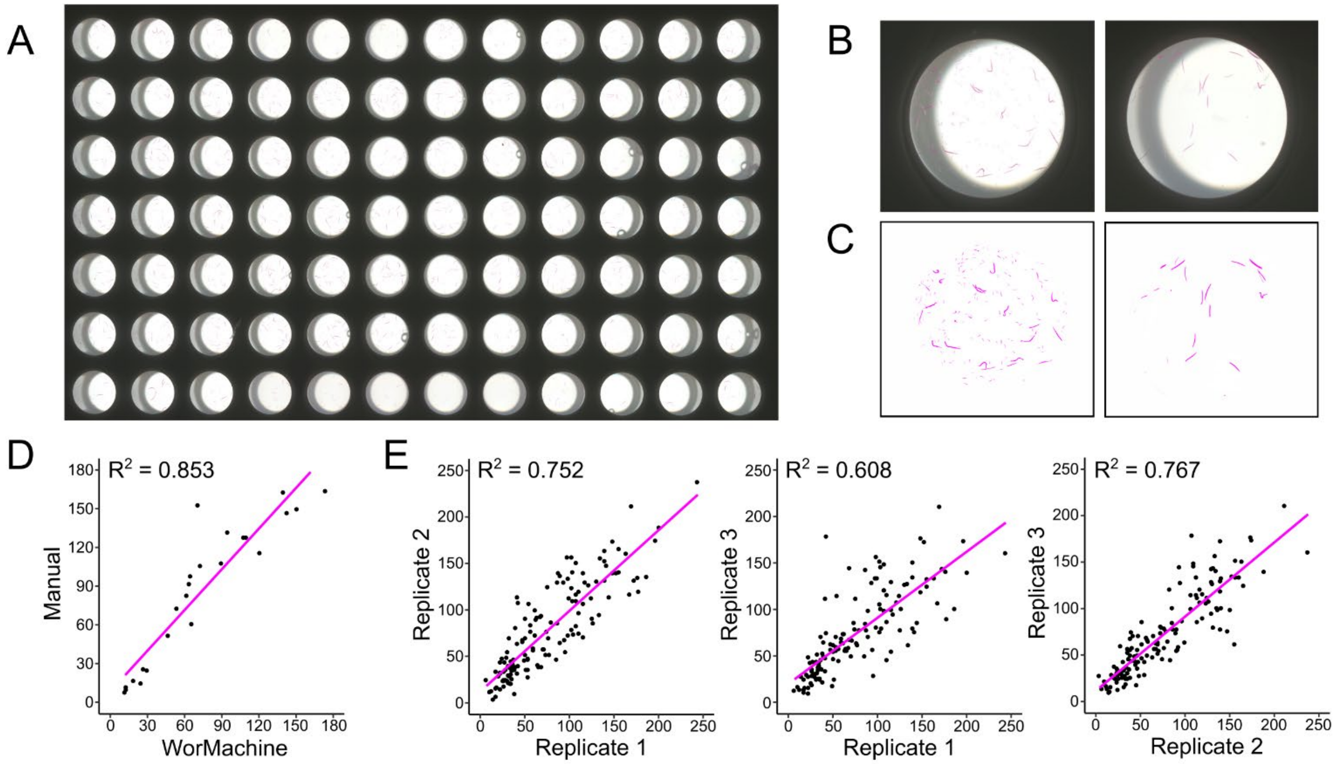

Figure S1. Quantitation of progeny number using semi-automated image processing. (A) Flatbed scanner image of a plate following staining and dilution steps. **(B)** Sample images of wells prior to processing. **(C)** Sample images of wells after processing. **(D)** Correlation between manual counts and WorMachine counts (N = 24). **(E)** Correlations between pairs of technical replicates as a measure of technical variability (N = 140 per correlation).

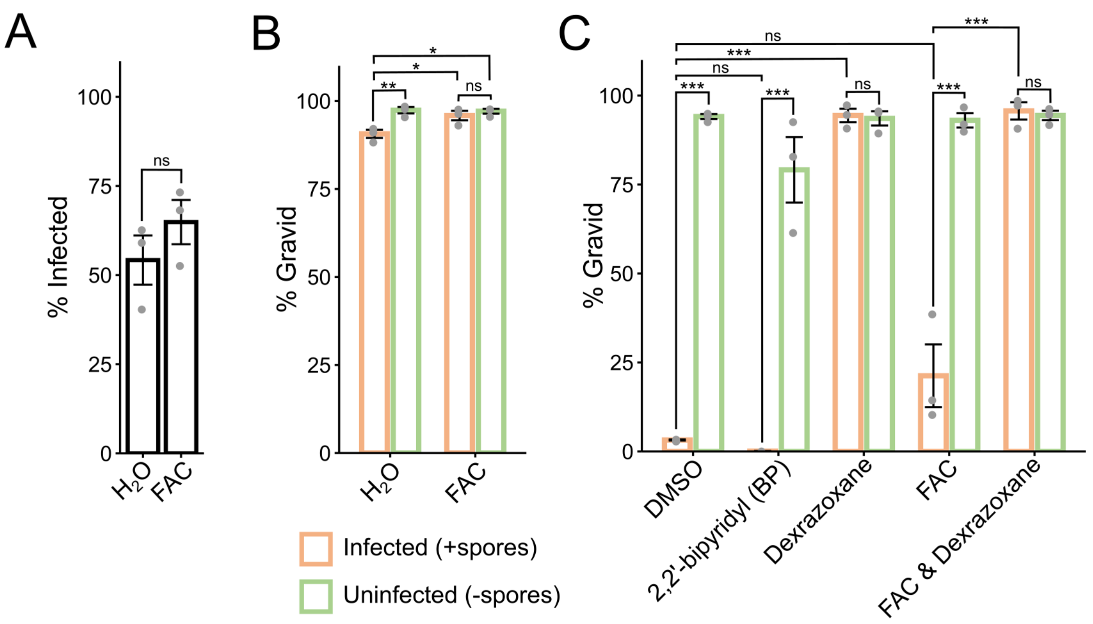

Figure S2. *N. parisii* infection in *C. elegans* is unaffected by altering iron levels. (A & B) Effects of iron supplementation with FAC on *N. parisii* infection in *C. elegans*. **(A)** Percentage of animals with newly formed spores (N = ≥140 animals counted per biological replicate; significance evaluated using a two-sample t-test) and **(B)** percentage of animals with embryos (N = ≥140 animals counted per biological replicate; significance evaluated using a one-way ANOVA with Tukey's post-hoc test) after 4 days of continuous infection with a low dose of spores are shown. **(C)** Effects of iron chelation with BP on infection, and effects of iron supplementation with FAC on dexrazoxane activity. Percentage of animals with embryos (N = ≥120 animals counted per biological replicate; significance evaluated using a one-way ANOVA with Tukey's post-hoc test) after 4 days of continuous infection with a normal dose of spores is shown. *** = *$p < 0.001$, ** = $p < 0.01$, * = $p < 0.05$, ns = not significant ($p > 0.05$)*. Data for each condition includes 3 biological replicates.

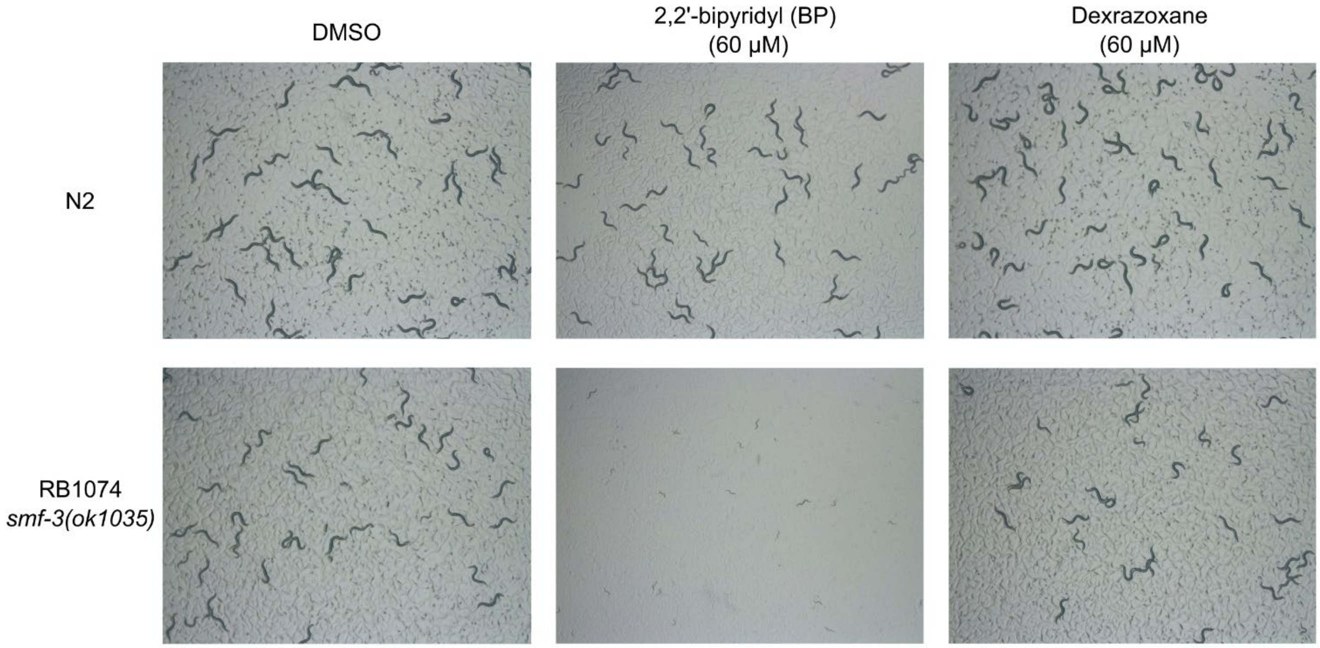

Figure S3. *C. elegans* mutant strain with reduced iron is not sensitive to dexrazoxane. RB1074 animals have 50% less iron compared to N2 and display severe growth defects during treatment with the iron chelator BP on NGM plates for 3 days from the L1 stage (bottom middle), but not after treatment with dexrazoxane (bottom right) (47).

Table S1: Compound names and available PubChem CIDs.

Compound	PubChem CID
Ethinyl Estradiol	5991
Menadione	4055
Parthenolide	108068
Plumbagin	10205
Curcumin	969516
Chloranil	8371
4,4'-Dimethoxydalbergione (Dalbergione)	364106
4,6-Dimethoxytoluquinone (Toluquinone)	NA
Thymoquinone	10281
1-Benzyloxycarbonylaminophenethyl chloromethyl ketone (ZPCK)	99625
Dexrazoxane	71384
